# Relationship between early-onset stroke and triglyceride-glucose index among young Chinese adults

**DOI:** 10.1186/s12944-023-01773-8

**Published:** 2023-01-11

**Authors:** Wenqi Xu, Haiyan Zhao, Xu Han, Jianrong Liu, Haixia Li, Junyan Sun, Aijun Xing, Shuohua Chen, Shouling Wu, Yuntao Wu

**Affiliations:** 1 Department of Cardiology, Kailuan General Hospital, Tangshan, 063000 China; 2 Graduate School, North China University of Science and Technology, Tangshan, China

**Keywords:** Triglyceride-glucose index, Early-onset stroke, Young Chinese adults

## Abstract

**Background:**

The triglyceride–glucose index (TyG index), an alternative indicator of peripheral insulin resistance (IR), is associated with cardiovascular disease (CVD) in the general population. The aim of this research was to determine the correlation between early-onset stroke and the TyG index among young Chinese adults.

**Methods:**

Participants (age ≤ 40 years) who attended their first physical examination in Kailuan General Hospital or its 11 subsidiary hospitals between 2006 and 2012 were enrolled. The subjects were divided into four equal points according to the quartile of the TyG index, with the lowest quartile (Q1) as the reference group. A Cox proportional hazard model was employed to assess the correlation between early-onset stroke incidence and the TyG index. Restricted cubic spline analysis was further conducted to examine nonlinear associations. The TyG index was calculated as Ln [Triglyceride (TG, mg/dL) × Fasting Blood Glucose (FBG, mg/dL)/2].

**Results:**

Overall, 35,999 subjects met the inclusion criteria. Their mean age was 30.8 ±  5.7 years, and 77.1% of subjects were males. During a median observation period of 11 years, 281 stroke events occurred (62 hemorrhagic strokes and 219 ischemic strokes). Compared to the Q1 group (as the lowest group), subjects in groups Q2-Q4 had significantly higher risks of early-onset stroke (*P* < 0.05) after adjustment for relevant confounders in the Cox proportional hazards model. Similar results were consistent with ischemic stroke. However, no significant associations were observed between the risk of hemorrhage and the baseline TyG index. The restricted cubic splines revealed that the risk of stroke progressively increased with a high TyG index ≥ 8.41.

**Conclusions:**

The TyG index may be a major risk factor for early-onset stroke among young Chinese adults. A TyG index ≥ 8.41 can be used as an indicator for screening high-risk stroke groups.

**Supplementary Information:**

The online version contains supplementary material available at 10.1186/s12944-023-01773-8.

## Background

Stroke is the most common cerebrovascular emergency in the general population and the leading cause of mortality and disability worldwide [1, 2]. The total number of disability-adjusted life years due to stroke has steadily increased since 1990, reaching 7.08 million deaths in 2020 (the number of deaths increased by 7.4% compared to 2019 [1, 3]. Moreover, the incidence of stroke among the younger population has also been steadily increasing; globally, the incidence rate of stroke in young people aged 15–49 has increased from 49.03/100,000–50.29/100,000 between 2016 and 2019 [3, 4]. In addition, the proportion of 15- to 49-year-old subjects among all persons with stroke has risen from 10% in 2016 to 16% in 2019 [3, 4]. Hypertension, diabetes, being overweight, obesity, and a poor lifestyle have been identified as the major risk factors related to stroke [5]. However, conventional risk factors do not entirely explain the increased stroke risk among young adults.

Insulin resistance (IR) has been confirmed as a dominant risk factor for diabetes, hypertension, and coronary heart disease [6–10]. In addition, epidemiological studies [11], animal studies [12], and clinical trials [13, 14] have all confirmed the association between IR and stroke. The hyperinsulinemic-euglycemic clamp (HEC) is regarded as the well-recognized gold standard for monitoring IR [15]. However, considering the economic benefits and clinical feasibility, clinical standardization has not yet been achieved. On the other hand, characteristics such as cost-effectiveness, simplicity, and efficiency make the triglyceride–glucose index (TyG index) a reliable surrogate for IR for preclinical and clinical applications [16, 17]. Furthermore, several studies confirmed the correlation between the TyG index and IR [18]. Previous studies within the general population have proposed that the TyG index is a major risk factor in predicting cardio-cerebrovascular disease [19–21]. However, as no previous study investigated the correlation between early-onset stroke and the TyG index among young Chinese adults, the aim of this research was to overcome this gap and address this issue.

## Methods

### Study subjects

The Kailuan Study is a multistage population-based cohort study whose aim is to investigate and intervene in the risk factors for cardiovascular and cerebrovascular diseases. Briefly, the cohort was initiated in July 2006, and examination rounds with registered employees and retirees were performed on average every 2 years at the Kailuan General Hospital and its 11 subsidiary hospitals. Currently, this project has completed seven follow-ups. Fasting Blood Glucose (FBG) and Triglyceride (TG) were measured at baseline and at each follow-up period. Since 2006, subjects have been followed up annually to evaluate the occurrence of cardiovascular disease (CVD) incidents, including stroke.

**Fig. 1:**
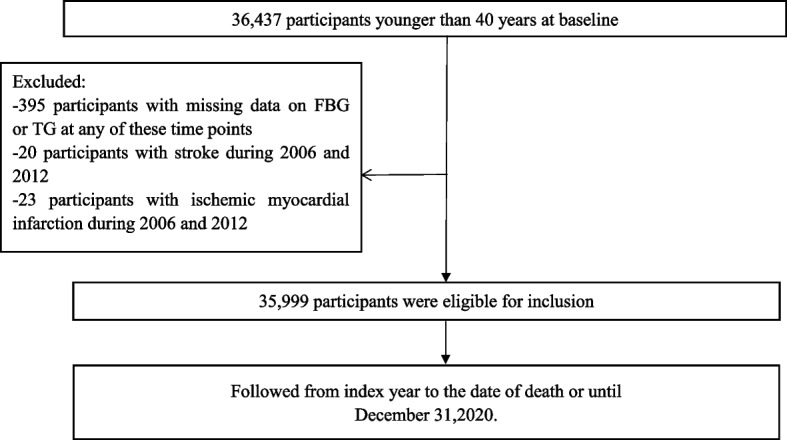
Flow chart for the inclusion of participants in the study

Individuals who attended the physical health examination for the first time in 2006–2012 and were ≤ 40 years old were selected as the research subjects. Participants with an obvious history of myocardial infarction (MI) or stroke was excluded. Additionally, those with missing data from FBG and TG data at baseline were excluded. The protocol for this study was in accordance with the Kailuan General Hospital Ethics Committee. All the subjects signed written informed consent before every survey circle (Fig. 1).

### Data collection

Baseline information on demographic characteristics (age and sex), physical examination (body weight and height, blood pressure), lifestyle characteristics (smoking habits, drinking habits, physical activity, salt intake, and educational level), history of the disease (stroke and MI), and medical history (the use of medication at baseline and during follow-up, such as antihypertensive agents, lipid-lowering agents, and hypoglycemic agents) were collected by experienced physicians in every circle. Hypertension was based on either receiving medications for hypertension, a self-reported history of diagnosed hypertension, or blood pressure ≥ 140/90 mmHg [22]. Diabetes status was based on receiving hypoglycemic drugs, a self-reported history of diagnosed diabetes, or FBG ≥ 7.0 mmol/L [23]. The remaining detailed design and basic description were obtained from published literature [24].

An equivalent of 5 mL peripheral venous blood sample was extracted from 7:00–9:00 am on the day of health examination after overnight (> 8 h) fasting. FBG was assessed using the hexokinase method with an upper limit of linearity of 33.3 mmol/L (coefficient of variation was < 2%). Serum TG was assessed using the enzymatic colorimetric method. Laboratory tests, such as high sensitivity C-reactive protein (hs-CRP), high-density lipoprotein cholesterol (HDL-C), total cholesterol (TC), and low-density lipoprotein cholesterol (LDL-C), were analyzed by an autoanalyzer (Hitachi, Tokyo, Japan). The corresponding kits were purchased from Zhongsheng North Control Biotechnology Co., Ltd (Peking, China). The professional quality controller regularly monitored these parameters.

### TyG index calculation

The TyG index was calculated as ln [TG (mg/dL) × FBG (mg/dL)/2] [16]. The subjects were classified into four categories based on the quartile of the TyG index: Q1 (6.38 ≤ TyG < 8.01), Q2 (8.01 ≤ TyG < 8.41), Q3 (8.41 ≤ TyG < 8.86), and Q4 (8.86 ≤ TyG < 14.20).

### Outcomes and follow-up

The starting point of follow-up was the date of completion of the physical health examination for the first time in 2006–2012; the endpoint of follow-up was either the first occurrence of stroke, the time of death, or the deadline of follow-up (December 31, 2020). Stroke is typically categorized as ischemic stroke, intracerebral hemorrhage, and subarachnoid hemorrhage, with the latter two being grouped into a hemorrhagic stroke. When combined with neuroimaging, stroke is generally defined in clinical practice as a syndrome of rapid onset characterized by focal cerebral dysfunction [25].

Specifically, ischemic stroke was defined as an acute neurological deficit confirmed cerebral infarction according to ICD-10 I63, and hemorrhagic stroke was defined as the acute extravasation of blood into the brain parenchyma and cerebral hemorrhage according to ICD-10 I61. If two or more events occurred, the first event was considered the primary endpoint. The data of all events were obtained through the Medicare system in Kailuan General Hospital. The endpoints and adverse events were recorded by trained medical personnel, and a physician confirmed all diagnoses.

### Statistical analyses

SAS 9.4 (SAS Institute Inc., Cary, NC, USA) was used for all analyses. The baseline data are displayed as the mean ± standard deviation (X ± SD), numbers (percentage), or medians (P25, P75), where appropriate. Different groups were compared using ANOVA, the Kruskal‒Wallis test, and the chi-square test. Data distributions were examined for normality using the Kolmogorov‒Smirnov test. The cumulative incidence of time to the event was graphically represented using Kaplan‒Meier curves, and the difference in each group was compared by the log-rank test.

The hazard ratios (HRs) and 95% CIs for stroke and subtypes were determined by a Cox proportional hazards analysis according to the TyG index. Competing risk regression analyses were conducted for the competing risk of death by considering the subdistribution hazard with nonstroke deaths as the competing risk [26]. Additionally, restricted cubic spline models were fitted to explore the shape of the correlation between the risk of early-onset stroke and baseline TyG index.

The researchers stratified the analyses of the baseline TyG index and incident early-onset stroke according to the following factors: alcohol consumption (never or ever/current), smoking (never or ever/current), physical activity (never or ever/current), hypertension (no or yes), diabetes (no or yes), and obesity [BMI < 28 or 28 kg/m^2^]. Considering that subarachnoid hemorrhage is less or differently associated with insulin resistance than other types of stroke, restricted analysis was performed by excluding participants with subarachnoid hemorrhage stroke in the hemorrhagic stroke group. Several sensitivity analyses were adopted to guarantee the robustness of the optimization results by excluding the participants who used antihypertensive, antiglycemic, and lipid-lowering drugs. Furthermore, we used the C-statistics index, integrated discrimination improvement (IDI), and net reclassification index (NRI) to compare the improvement in the predictive value of the TyG index and LDL-C for stroke beyond traditional risk factors. A two-tailed *P* value was set at 0.05.

## Results

### Characteristics of the study population

Among 35999 participants, 77.10% were men, and their mean age was 30.8 ± 5.7 years. The median (IQR) of the baseline TyG index was 8.41 (8.48 ± 0.68). The clinical parameters of the study subjects, according to quartiles of the TyG index, are listed in Table 1. Subjects in the elevated TyG index group were more inclined to be current alcohol drinkers and smokers, with higher levels of hs-CRP, FBG, TG, and LDL-C. They also had higher proportions of diabetes and hypertension. A statistically significant between-group difference was recorded (*P* < 0.05).

**Table 1:** Baseline characteristics according to quartiles of baseline TyG index

	**Total**	**Quartile 1**	**Quartile 2**	**Quartile3**	**Quartile 4**	***P***
Participants	35,999	9019	9015	8972	8993	
Age, year	30.82 ± 5.66	29.78 ± 5.64	30.37 ± 5.66	30.94 ± 5.65	32.19 ± 5.41	< 0.01
Male, N (%)	27,744(77.10)	5340(59.20)	6674(74.00)	7431(82.80)	8299(92.30)	< 0.01
SBP, mmHg	118.18 ± 14.65	112.78 ± 13.07	116.14 ± 13.55	119.67 ± 14.33	124.19 ± 15.09	< 0.01
DBP, mmHg	78.58 ± 10.02	74.90 ± 8.86	77.13 ± 9.24	79.51 ± 9.70	82.77 ± 10.48	< 0.01
TyG index	8.48 ± 0.68	7.69 ± 0.25	8.22 ± 0.11	8.62 ± 0.13	9.38 ± 0.47	< 0.01
BMI, kg/m^2^	24.35 ± 3.88	22.38 ± 3.26	23.59 ± 3.51	24.87 ± 3.65	26.58 ± 3.77	< 0.01
FBG, mmol/L	5.08 ± 0.87	4.77 ± 0.54	4.93 ± 0.57	5.13 ± 0.63	5.50 ± 1.32	< 0.01
TC, mmol/L	4.61 ± 0.97	4.22 ± 0.78	4.49 ± 0.81	4.73 ± 0.87	4.98 ± 1.20	< 0.01
LDL-C, mmol/L	2.40 ± 0.73	2.16 ± 0.70	2.40 ± 0.67	2.52 ± 0.69	2.51 ± 0.80	< 0.01
HDL-C, mmol/L	1.42 ± 0.34	1.47 ± 0.35	1.43 ± 0.32	1.41 ± 0.33	1.37 ± 0.37	< 0.01
HR, bpm	73.73 ± 9.75	72.77 ± 9.56	73.07 ± 9.69	73.86 ± 9.67	75.22 ± 9.90	< 0.01
TG, mmol/L	1.13(0.77–1.72)	0.60(0.50–0.69)	0.96(0.85–1.06)	1.36(1.21–1.53)	2.51(2.00–3.51)	< 0.01
hs-CRP, mg/L	1.00(0.40–2.43)	0.83(0.33–2.06)	1.00(0.40–2.41)	1.10(0.49–2.40)	1.24(0.55–2.75)	< 0.01
Current smoking, N (%)	13,335(37.00)	2594(28.80)	3017(33.50)	3424(38.20)	4300(47.80)	< 0.01
Current drinking, N (%)	14,051(39.00)	2833(31.40)	3130(34.70)	3625(40.40)	4463(49.60)	< 0.01
Physical exercisers, N (%)	29,319(81.40)	7345(81.40)	7449(82.60)	7364(82.10)	7161(79.60)	< 0.01
Education level, N (%)						< 0.01
Highschool diploma or below	15,948(44.30)	3436(38.10)	3888(43.10)	4141(46.20)	4483(49.80)	
Highschool diploma or above	20,051(55.70)	5583(61.90)	5127(56.90)	4831(53.80)	4510(50.20)	
Salt level, g/d						< 0.01
< 6	4199(11.70)	1137(12.60)	988(11.00)	1054(11.70)	1020(11.30)	
6–10	27,823(77.30)	6939(76.90)	7144(79.20)	6928(77.20)	6812(75.70)	
Diabetes, N (%)	740(2.06)	24(0.27)	40(0.44)	101(1.13)	575(6.39)	< 0.01
Antidiabetic treatment, N (%)	64(0.18)	3(0.03)	10(0.11)	14(0.16)	37(0.41)	< 0.01
Hypertension, N (%)	6367(17.70)	644(7.14)	1136(12.6)	1806(20.1)	2781(30.9)	< 0.01
Lipid-lowering treatment, N (%)	69(0.19)	6(0.07)	13(0.14)	9(0.10)	41(0.46)	< 0.01
Antihypertensive treatment, N (%)	639(1.78)	67(0.74)	86(0.95)	166(1.85)	320(3.56)	< 0.01

*P*, comparison according to quartiles of baseline TyG index

*TyG index* triglyceride-glucose index, *SBP* systolic blood pressure, *DBP* diastolic blood pressure, *BMI* body mass index, *HR* heart rate, *TG* triglyceride, *HDL-C* high-density lipoprotein cholesterol, *LDL-C* low-density lipoprotein cholesterol, *FBG* fasting blood glucose, *hs-CRP* high-sensitivity C reactive protein

### Characteristics of stroke and subtypes

**Fig. 2:**
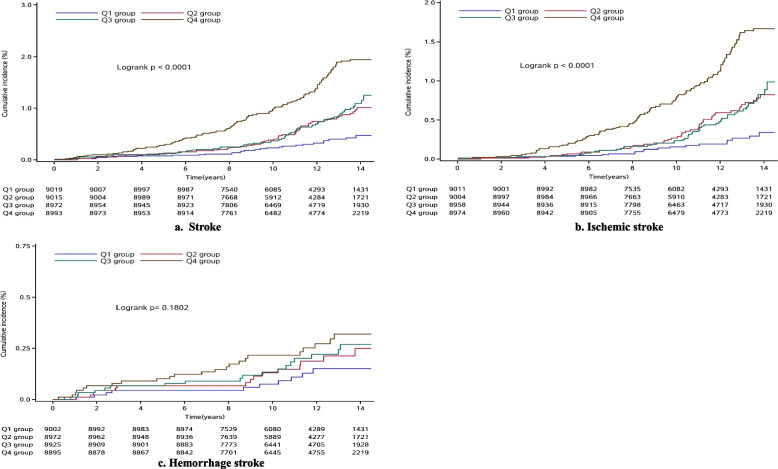
Cumulative incidence according to quartiles of baseline TyG index. **a** Stroke; **b** Ischemic stroke; **c** Hemorrhage stroke

The length of the follow-up period was 11.50 ± 2.61 years. During this period, 281 (0.78%) participants suffered a stroke, 219 of whom had an ischemic stroke, and the remaining had a hemorrhagic stroke. The first stroke incidence occurred at a mean ± SD age of 43.8 ± 6.3 years. The incidence rates of stroke events were 0.27, 0.57, 0.64, and 1.22/1000 person-years for the Q1-Q4 groups, respectively (Table 2). Concurrently, the incidence rates of ischemic stroke were 0.19, 0.45, 0.47, and 1.02/1000 person-years for the Q1-Q4 groups, respectively (Table 2). Thus, there was a tendency toward an increased risk of stroke and its subtypes among groups. The cumulative incidence of stroke and its subtypes for each of the TyG index quartiles was calculated using the log-rank test (*P* < 0.05). However, no apparent difference was detected in hemorrhagic stroke between groups (*P* > 0.05, Fig. 2).

**Table 2:** HRs for risk of outcomes according to quartiles of baseline TyG index

	**Quartile 1**	**Quartile 2**	**Quartile 3**	**Quartile 4**	***P* for trend**
**Stroke, N (%)**	28(0.31)	58(0.64)	67(0.75)	128(1.42)	
Incidence, per1000 person-y	0.27	0.57	0.64	1.22	
Model 1	Reference	1.70(1.08–2.68)	1.62(1.04–2.53)	2.50(1.64–3.81)	< 0.01
Model 2	Reference	1.65(1.05–2.60)	1.49(0.95–2.34)	2.11(1.38–3.24)	0.01
Model 3	Reference	1.57(1.00–2.47)	1.35(0.86–2.12)	1.78(1.15–2.74)	0.02
**Ischemic stroke, N (%)**	19(0.21)	46(0.51)	49(0.55)	107(1.19)	
Incidence, per1000 person-y	0.19	0.45	0.47	1.02	
Model 1	Reference	1.96(1.14–3.37)	1.70(0.98–2.93)	2.96(1.76–4.96)	< 0.01
Model 2	Reference	1.89(1.10–3.25)	1.54(0.89–2.64)	2.40(1.43–4.03)	0.01
Model 3	Reference	1.78(1.03–3.07)	1.36(0.79–2.36)	1.98(1.17–3.35)	0.02
**Hemorrhage stroke, N (%)**	10(0.11)	15(0.17)	18(0.20)	23(0.26)	
Incidence, per1000 person-y	0.10	0.15	0.17	0.22	
Model 1	Reference	1.26(0.57–2.83)	1.28(0.59–2.81)	1.37(0.63–2.95)	0.46
Model 2	Reference	1.24(0.56–2.78)	1.24(0.56–2.73)	1.31(0.60–2.88)	0.54
Model 3	Reference	1.23(0.55–2.76)	1.16(0.52–2.58)	1.14(0.51–2.54)	0.86

Model 1: adjusted for age and sex; Model 2: adjusted for age, sex, smoking, drinking, education level, salt status, physical activity, and BMI; Model 3: adjusted for all the variables in model 2 and LDL-C, HDL-C, hs-CRP, hypertension, antidiabetic drugs, antihypertensive drugs and lipid-lowering drugs. TyG indicates triglyceride-glucose

### Association of stroke and TyG index

Taking into account multiple potential confounders, data were adjusted for sex, age, salt intake, physical activity, education level, alcohol abuse, smoking status, hs-CRP, HDL-C, LDL-C, systolic blood pressure (SBP), diastolic blood pressure (DBP), BMI, history of antidiabetic drugs, lipid-lowering drugs, antihypertensive drugs, and hypertension. The primary result of Cox regression analysis showed that compared to the TyG index level in Q1, the adjusted HRs (95% CI) for stroke in Q2- Q4 were 1.57 (1.00–2.47), 1.35 (0.86–2.12), and 1.78 (1.15–2.74), respectively. With increasing TyG index quartiles, multivariable-adjusted HRs (95% CI) for ischemic stroke within the Q2-Q4 groups were 1.78 (1.03–3.07), 1.36 (0.79–2.36), and 1.98 (1.17–3.35), respectively. However, there was a nonsignificant association between hemorrhagic stroke and the baseline TyG index (Table 2). Essentially similar results were revealed according to the competing risk analyses (Table 3).

**Table 3:** HRs from Competing Risk Models for outcomes according to quartiles of baseline TyG index

	**Total**	**Quartile 1**		**Quartile 2**	**Quartile 3**	**Quartile 4**	***P* for trend**
**Strok, N (%)**	281		28(0.31)	58(0.64)	67(0.75)	128(1.42)	
Model 1			Reference	1.70(1.08–2.69)	1.62(1.03–2.54)	2.49(1.62–3.84)	< 0.01
Model 2			Reference	1.65(1.04–2.60)	1.49(0.95–2.34)	2.11(1.36–3.25)	0.01
Model 3			Reference	1.57(0.99–2.49)	1.34(0.85–2.12)	1.78(1.14–2.76)	0.02
**Ischemic stroke, N (%)**	221		19(0.21)	46(0.51)	49(0.55)	107(1.19)	
Model 1			Reference	1.96(1.14–3.37)	1.70(0.98–2.93)	2.96(1.70–4.96)	< 0.01
Model 2			Reference	1.89(1.10–3.25)	1.54(0.89–2.64)	2.40(1.43–4.03)	0.01
Model 3			Reference	1.78(1.03–3.07)	1.36(0.79–2.36)	1.98(1.17–3.37)	0.03
**Hemorrhage stroke, N (%)**	66		10(0.11)	15(0.17)	18(0.20)	23(0.26)	
Model 1			Reference	1.26(0.56–2.85)	1.28(0.58–2.81)	1.36(0.63–2.95)	0.46
Model 2			Reference	1.24(0.55–2.79)	1.24(0.55–2.76)	1.31(0.60–2.88)	0.55
Model 3			Reference	1.22(0.53–2.80)	1.16(0.51–2.61)	1.14(0.51–2.52)	0.86

Model 1: adjusted for age and sex;

Model 2: adjusted for age, sex, smoking, drinking, education level, salt status, physical activity, and BMI;

Model 3: adjusted for all the variables in model 2 and LDL-C, HDL-C, hs-CRP, hypertension, antidiabetic drugs, antihypertensive drugs and lipid-lowering drugs

**Fig. 3:**
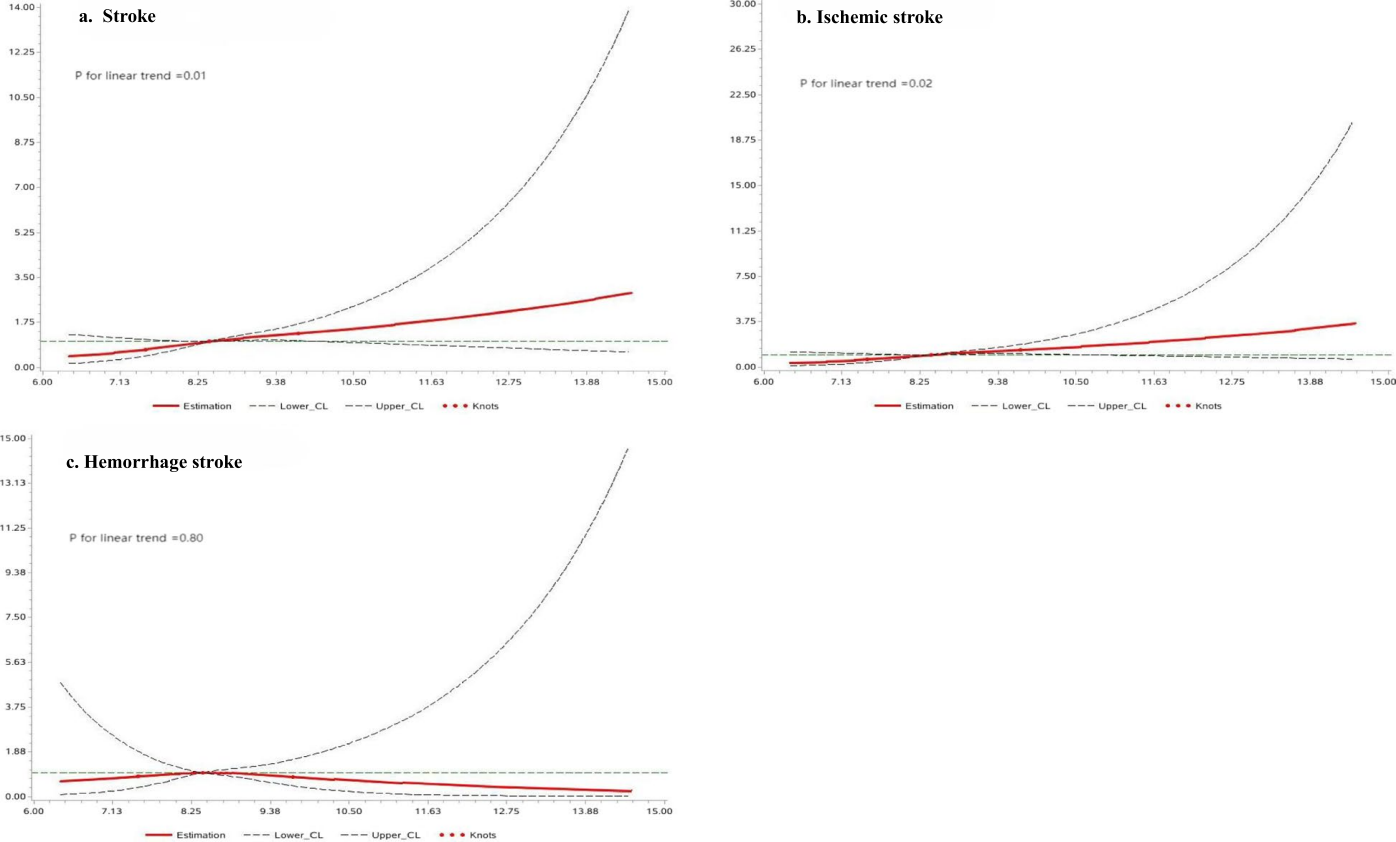
Adjusted hazard ratios of outcomes according to baseline TyG index. **a** Stroke; **b** ischemic stroke; **c** hemorrhage stroke. **a-c** adjusted for age, sex, smoking, drinking, education level, salt status and physical activity, BMI, LDL-C, HDL-C, hs-CRP, hypertension, antidiabetic drugs, antihypertensive drugs and lipid-lowering drugs; Data were fitted using a Cox regression model of restricted cubic spline with three knots (at the 5th, 50th, and 95th percentiles). Adjusting for potential covariates. The reference point for the TyG index was the median of the reference group. Red lines indicate adjusted hazard ratios, and black lines indicate the 95% confidence interval bands

After adjusting for confounding factors, the results from restricted cubic splines revealed a linear association of stroke and ischemic stroke with the TyG index (*P* linearity = 0.02, 0.01; *P* nonlinearity = 0.49, 0.44). Moreover, when the TyG index was ≥ 8.41, the risk of stroke started to increase with the rising TyG index (Fig. 3).

### Subgroup analysis

In addition, stratified analyses were performed to identify potentially confounding factors. No significant interactions were detected between potential risk factors, such as drinking, smoking, physical exercise, obesity, hypertension, and diabetes, and the TyG index for the risk of early-onset stroke. Using the lowest quartile group (Q1) as a reference, significant positive associations were observed for nondrinkers, nonsmokers, physical activity, nonhypertension, and nondiabetes groups; the HRs (95% CI) were 2.57 (1.32–5.01), 2.07 (1.12–3.81), 2.09 (1.27–3.43), 2.03 (1.18–3.49), and 1.84 (1.19–2.85), respectively. However, no statistically significant differences were found in the drinking, smoking, no physical activity, hypertension, and diabetes groups (Table S1).

### Sensitivity analyses

The subjects on antihypertensive medications, anti-glycemic medications, and lipid-lowering medications at baseline and the duration of follow-up were excluded, and the analysis using a Cox regression-based model was repeated. The results were concordant with the primary findings. Consequently, no significant change was found in the relationship of outcomes and the TyG index, indicating the robustness of this finding (Table S2).

Considering that subarachnoid hemorrhage is usually caused by inherited vascular malformations, restricted analysis was performed by excluding subarachnoid hemorrhage stroke in the hemorrhagic stroke group. All generated similar findings to the primary analysis. In addition, the results became more significant compared with the primary analysis (Table S3-4).

### Incremental predictive value of the TyG index

To examine whether the TyG index could have a superior predictive value of the conventional risk factors for stroke compared with other markers of atherosclerosis, such as LDL-C, the C statistic, IDI and NRI were calculated (Table 4). The C index significantly improved after the addition of the TyG index (from 0.8035 to 0.8078, *P* < 0.05), and the discriminatory power and risk reclassification were also substantially better, with an IDI of 0.0007 (95% CI, 0–0.0015) and an NRI of 0.1381 (95% CI, 0.0184–0.2579). Compared with LDL-C, the C index, IDI and NRI were all more improved after adding the TyG index to the conventional model.

**Table 4:** Incremental ability of the TyG index and LDL-c to predict stroke

	**C-index**		**IDI**		**Category-free NRI**	
	Est. (95% CI)	*P*	Est. (95% CI)	*P*	Est. (95% CI)	*P*
Clinical risk factors	0.8035 (0.7798–0.8272)	-	-	-	-	-
Clinical risk factors + TyG index	0.8078 (0.7844–0.8311)	0.05	0.0007 (0–0.0015)	0.05	0.1381 (0.0184–0.2579)	0.02
Clinical risk factors + LDL-c	0.8043 (0.7807–0.8279)	0.19	0.0001 (0–0.0002)	0.63	0.0391 (0.0802–0.1584)	0.52

Clinical risk factors: age, sex, smoking, drinking, education level, salt status, physical activity, BMI, HDL-C, hs-CRP, antidiabetic drugs, antihypertensive drugs and lipid-lowering drugs

TyG indicates triglyceride-glucose

## Discussion

The TyG index (as the surrogate marker of IR) might be considered a significant risk factor for early-onset stroke among young adults, particularly ischemic stroke. Moreover, the association of early-onset stroke with the TyG index had a linear dose‒response relationship independent of other conventional risk factors. In addition, the association was more significant in participants without traditional risk factors, such as drinking, smoking, no physical exercise, hypertension, and diabetes, than in those with traditional risk factors. Furthermore, the addition of the TyG index to the conventional risk model, which is considered an important potential predictor, significantly promoted the risk stratification ability.

During the 11-year follow-up period, the future risk of early-onset stroke was found to be positively related to the baseline TyG index. Compared to the Q1 group, the TyG index in the Q4 group had a 78% higher risk for incident early-onset stroke. Notably, after adjusting the results for the competing risk analysis and the impact of taking antihypertensive, antiglycemic, and lipid-lowering medications, the relationship of early-onset stroke and the TyG index remained consistent with the primary outcome. While no previous study has discussed the correlation between early-onset stroke and TyG index among young individuals, a collaborative meta-analysis of 8 prospective studies found that ischemic stroke was more likely to occur in people with high TyG indexes. Compared with subjects in the bottom quartiles of the TyG index, these findings suggested that subjects in the upper quartiles had a 1.26-fold increased risk of stroke [27]. In their study, Wang et al*.* [21] also demonstrated that the positive correlation between the occurrence of stroke and TyG index among the 18- to 98-year-old general population was positively associated with the presence of stroke. Compared to subjects in the lowest quartile (3.61 ≤ TyG < 8.18), the risk of stroke in the highest quartile group (9.05 ≤ TyG < 12.5) was increased by 32%. Although the risk values varied, the positive correlation tendency was consistent with this finding.

After stratification by traditional risk factors, no interaction was detected between the relative risk of early-onset stroke and the TyG index. The hierarchical analyses showed an association between early-onset stroke and a high TyG index in the nondrinking, nonsmoking, physical exercise, nonhypertension, and nondiabetic groups in the low-risk population. Conversely, no association was observed in the high-risk population with traditional risk factors. These results strongly suggested that the TyG index was correlated with a high risk of early-onset stroke among a low-risk youth population. Yang et al*.* [28] demonstrated that this positive association was pronounced in the population with nontraditional risk factors, which was further supported by the present findings. Given the age of the population, youth adults without traditional risk factors had a lower 10-year atherosclerotic disease (ASCVD) risk, while the young population had a lower risk [29]. Interestingly, the current results showed that the TyG index was a dominant independent factor for stroke in the low-risk young population. Thus, the current findings further the understanding of the risk factors for early-onset stroke in young adults. Moreover, the TyG index could also serve as a potential and new indicator for screening high-risk young individuals. Typically, when the TyG index was > 8.41, a strong correlation was established between the TyG index and early-onset stroke, requiring timely clinical intervention.

Previous studies demonstrated that using anti-hypertension [30], lipid-lowering [31], and hypoglycemic medications [32] could modify the effect on the risk of stroke. To exclude the effect of hypoglycemic, anti-hypertension, and lipid-lowering drugs as confounding factors, the researchers undertook several sensitivity analyses; however, the results revealed no effect of any of the above-listed confounders. Nonetheless, the risk of stroke could not be decreased by eliminating the associated risk factors. These findings suggested that the TyG index acts as a robust risk factor among young adults, irrespective of the other risk factors.

Of note, LDL-c, which is a recognized marker of atherosclerosis, has a significant impact on the occurrence and development of stroke [33]. In contrast, compared to LDL-c, the addition of the TyG index to the conventional risk model has a more incremental effect on the predicted value for incident stroke. The predictive utility of the TyG index value for various cardiovascular event predictions was evaluated in previous studies, but the results were significantly inconsistent. The Tehran Lipid and Glucose Study revealed that adding the TyG index to the Framingham model does not provide greater risk prediction for CVD [34]. However, in most other research, such as the analysis of the CARDIA study, which adding the TyG index to the PCEs model can improve the predictive ability for CVD events [35]. This was basically in accordance with the analysis results of this research. Thus, in the present study, we validated that the TyG index has better clinical predictive power for early-onset stroke among young adults. This finding highlights the importance of monitoring the dynamic TyG index in clinical practice.

Although researchers have found a strong correlation between the risk of early-onset stroke and IR assessed by the TyG index, there is still no clear understanding of the underlying mechanism. Previous studies have proposed several potential causal pathways. First, since hyperinsulinemia accompanies IR, it may result in sympathetic activation, as well as increased tubular sodium reabsorption and endothelin secretion, all of which favor a rise in blood pressure [36, 37]. The potential underlying mechanisms may also involve excessive inflammation and oxidative stress, subsequently causing vascular endothelial function [9, 38, 39]. Second, IR may cause lipoprotein abnormalities, including hypertriglyceridemia, a decrease in HDL, and a change in LDL particle size, thus further aggravating IR. Plaques are regarded as a high-risk factor during the occurrence of stroke. Lipoprotein abnormalities and endothelial dysfunction may accelerate the transformation of macrophages into foam cells, which are the key components of the pathogenesis of stroke. Additionally, IR is linked to diminished fibrinolysis and substantial platelet aggregation, which obstruct cerebral arteries and result in hemodynamic abnormalities and the creation of thrombi [40].

Consistent with previous studies [21], the TyG index has been independently corroborated as a key risk factor for stroke. This study has expanded the existing knowledge on the TyG index and stroke. To the best of our knowledge, this is the first prospective study in China that examined the association between early-onset stroke and the TyG index. Furthermore, this is also the first study that established the optimal threshold of the TyG index for early-onset stroke. The results underscore the importance of keeping the TyG index below the threshold among young adults. The findings of this study have major public health and clinical implications. The current study confirmed that the TyG index is correlated with the relative risk of early-onset stroke among young Chinese adults, irrespective of the burden of traditional risk factors. This finding provides a new basis for preventing early-onset stroke in the young population. Furthermore, the cutoff of the TyG index for identifying stroke was not determined. The results implied that when the TyG index is ≥ 8.41, the risk of stroke increases with a high TyG index. Thus, the researchers recommend a threshold point of 8.14 for the TyG index to identify young adults at future risk of stroke. Supposedly, early intervention is critical, and treatment should be administered when the TyG index is > 8.14. Lifestyle interventions, such as physical exercise and weight loss, remain the foundation of primary stroke prevention and have a significant desirable effect on the TyG index. Moreover, lipid-lowering drugs, including niacin and fibrates, should be administered to strengthen the lipid-lowering therapy that reverses poor outcomes.

### Study strengths and limitations

The present study has several strengths. This research was based on a large community-based cohort design used to evaluate the correlation between early-onset stroke and the TyG index among young Chinese adults through extensive prospective data with up to 11 years of follow-up. Additionally, information about all participants, including whether a clinical stroke occurred and the exact time of events that took place, were carefully recorded to ensure tracked accuracy and quality data. The present study also has some limitations. First, limited by human, material, and financial resources and other factors, and due to the absence of fasting insulin measurement and nonevaluation of HOMA-IR in the Kailuan Study [41], it was impossible to compare the differences between HOMA-IR and TyG index on early-onset stroke. Second, participants included the typical Chinese population from northern China, most of whom were adult community-dwelling men in a middle city in northern China. Although the subjects of this study comprised a young occupational population in northern China, the reported results still have a significant reference value because the incidence density of this study was not in contradiction with the previous reports (0.53/1000 person-years and 0.76/1000 person-years, respectively) [42, 43]. Third, no lacunar stroke subjects included in this cohort contributed to a decrement in the outcome of ischemic stroke, which could underestimate the influences of the TyG index on adverse outcomes. Fourth, given the inherent nature of observational studies, a causal relationship between the TyG index and early-onset stroke development could not be determined. Fifth, although adjusting for multiple variables in the Cox regression model, residual confounding factors could potentially exist, including behavioral and environmental factors. Finally, further studies are needed to explore the long-term effect of the dynamic TyG index on progressive stroke by using other methods, such as trajectory analysis.

## Conclusion

Overall, the present study supports the evident association between early-onset stroke and the TyG index among young Chinese adults, especially ischemic stroke. Furthermore, the researchers provided reference ranges in the risk threshold of the TyG index (TyG = 8.41). Thus, the TyG index may serve as a practical tool for early-onset stroke risk assessment in clinical applications. A TyG index > 8.41 may be applied as a screening index to identify the high-risk population. Furthermore, timely emphasis on the primary prevention of the TyG index provides an opportunity for preventing or delaying early-onset stroke.

## Supplementary Information

**Additional file 1: Supplementary Table 1.** Stratification analysis of HRs for risk of outcomes according to quartiles of baseline TyG index. **Supplementary Table 2.** Sensitive analysis for hazard ratios values and 95% Confidence Intervals (CI) according to the period of baseline and follow-up. **Supplementary Table 3.** HRs for risk of outcomes after excluding the participants with subarachnoid hemorrhage stroke(*n*=11). **Supplementary Table 4.** HRs from Competing Risk Models for outcomes after excluding the participants with subarachnoid hemorrhage stroke(*n*=11).

## Data Availability

The datasets during and/or analyzed during the current study available from the corresponding author on reasonable request.

## Abbreviations

TyG: Triglyceride-glucose
CVD: Cardiovascular disease
TG: Triglyceride
FBG: Fasting blood glucose
IR: Insulin resistance
HEC: Hyperinsulinemic-euglycemic clamp
MI: Myocardial infarction
HDL-C: High-density lipoprotein cholesterol
LDL-C: Low-density lipoprotein cholesterol
hs-CRP: High-sensitivity C-reactive protein
TC: Total cholesterol
SBP: Systolic blood pressure
DBP: Diastolic blood pressure
CI: Confidence interval
