# Supplementary material for: Relationship between early-onset stroke and triglyceride-glucose index among young Chinese adults
Source: Lipids Health Dis. 2023 Jan 11;22:3. doi: 10.1186/s12944-023-01773-8 (PMC9832803; doi:10.1186/s12944-023-01773-8)
Supplement: Supplementary file 1 — Additional file 1: Supplementary Table 1. Stratification analysis of HRs for risk of outcomes according to quartiles of baseline TyG index. Supplementary Table 2. Sensitive analysis for hazard ratios values and 95% Confidence Intervals (CI) according to the period of baseline and follow-up. Supplementary Table 3. HRs for risk of outcomes after excluding the participants with subarachnoid hemorrhage stroke(n=11). Supplementary Table 4. HRs from Competing Risk Models for outcomes after excluding the participants with subarachnoid hemorrhage stroke(n=11). [file 12944_2023_1773_MOESM1_ESM.docx]

**Supplementary** **Table1** Stratification analysis of HRs for risk of outcomes according to quartiles of baseline TyG index

| **Outcomes** |  |  |  | **TyG index** | | | | ***P* for interaction** |
| --- | --- | --- | --- | --- | --- | --- | --- | --- |
|  | **N** | **Case** | **Incidence (%)** | **Quartile 1** | **Quartile 2** | **Quartile 3** | **Quartile 4** |  |
| Total | 35999 | 281 | 0.78 |  |  |  |  |  |
| **Smoking** |  |  |  |  |  |  |  | 0.86 |
| Never/Ever | 22664 | 144 | 0.64 | Reference | 1.80（0.97-3.33） | 1.41（0.75-2.65） | 2.07（1.12-3.81） |  |
| Current | 13335 | 137 | 1.03 | Reference | 1.31（0.67-2.58） | 1.25（0.65-2.40） | 1.45（0.79-2.69） |  |
| **Alcohol drinking** |  |  |  |  |  |  |  | 0.20 |
| Never/Ever | 21948 | 136 | 0.62 | Reference | 2.29（1.18-4.45） | 1.51（0.75-3.02） | 2.57（1.32-5.01） |  |
| Current | 14051 | 145 | 1.03 | Reference | 1.06（0.56-2.01） | 1.22（0.67-2.21） | 1.26（0.72-2.23） |  |
| **Physicial activity** |  |  |  |  |  |  |  | 0.35 |
| Never/Ever | 6680 | 51 | 0.76 | Reference | 1.08（0.42-2.83） | 1.04（0.41-2.65） | 0.83（0.33-2.11） |  |
| Current | 29319 | 230 | 0.78 | Reference | 1.70（1.01-2.85） | 1.43（0.85-2.39） | 2.09（1.27-3.43） |  |
| **Obesity** |  |  |  |  |  |  |  | 0.81 |
| BMI<28kg/m² | 29887 | 190 | 0.64 | Reference | 1.40(0.87-2.28) | 1.26（0.77-2.04） | 1.53（0.95-2.46） |  |
| BMI≥28kg/m² | 6112 | 91 | 1.49 | Reference | 2.84(0.63-12.86) | 2.07（0.48-8.98） | 3.13（0.76-12.94） |  |
| **Hypertention** |  |  |  |  |  |  |  | 0.33 |
| No | 29632 | 153 | 0.52 | Reference | 1.83（1.06-3.14） | 1.84（1.07-3.16） | 2.03（1.18-3.49） |  |
| Yes | 6367 | 128 | 2.01 | Reference | 1.22（0.53-2.80） | 0.89（0.40-1.98） | 1.53（0.73-3.20） |  |
| **Diabetes** |  |  |  |  |  |  |  | 0.99 |
| No | 35259 | 258 | 0.73 | Reference | 1.66（1.05-2.62） | 1.45（0.93-2.28） | 1.84（1.19-2.85） |  |
| Yes | 740 | 23 | 3.10 | Reference | 0.49（0.18-1.29） | 0.39（0.15-1.01） | 0.90（0.38-2.14） |  |

Model 1: adjusted for age and sex;

Model 2: adjusted for age, sex, smoking, drinking, education level, salt status, physical activity, and BMI;

Model 3: adjusted for all the variables in model 2 and LDL-C, HDL-C, hs-CRP, hypertension, antidiabetic drugs, antihypertensive drugs and lipid-lowering drugs.

TyG indicates triglyceride-glucose.

| **Supplementary** **Table2** Sensitive analysis for hazard ratios values and 95% Confidence Intervals (CI) according to the period of baseline and follow-up | | | | | |
| --- | --- | --- | --- | --- | --- |
|  | **Quartile 1** | **Quartile 2** | **Quartile 3** | **Quartile 4** | ***P* for trend** |
| **Without Antihypertensive treatment** |  |  |  |  |  |
| **stroke** |  |  |  |  |  |
| Model 1 | Reference | 1.59（0.96-2.64） | 1.57（0.95-2.59） | 2.32（1.45-3.75） | 0.01 |
| Model 2 | Reference | 1.54（0.93-2.56） | 1.40（0.85-2.33） | 1.89（1.16-3.07） | 0.02 |
| Model 3 | Reference | 1.48（0.89-2.47） | 1.29（0.78-2.16） | 1.66（1.02-2.73） | 0.08 |
| **Ischemic stroke** |  |  |  |  |  |
| Model 1 | Reference | 1.98（1.06-3.07） | 1.72（0.92-3.23） | 3.02（1.68-5.45） | 0.01 |
| Model 2 | Reference | 1.89（1.01-3.55） | 1.49（0.79-2.82） | 2.32（1.27-4.23） | 0.05 |
| Model 3 | Reference | 1.80（0.96-3.37） | 1.35（0.71-2.57） | 2.02（1.10-3.71） | 0.05 |
| **Hemorrhage stroke** |  |  |  |  |  |
| Model 1 | Reference | 1.13（0.49-2.59） | 1.18（0.52-2.65） | 1.07（0.47-2.45） | 0.02 |
| Model 2 | Reference | 1.11（0.49-2.56） | 1.12（0.50-2.54） | 0.99（0.43-2.30） | 0.95 |
| Model 3 | Reference | 1.09（0.47-2.52） | 1.04（0.45-2.39） | 0.86（0.36-2.03） | 0.66 |

| **Continue Supplementary** **Table2** |  |  |  |  |  |
| --- | --- | --- | --- | --- | --- |
|  | **Quartile 1** | **Quartile 2** | **Quartile 3** | **Quartile 4** | ***P* for trend** |
| **Without Antidiabetic treatment** |  |  |  |  |  |
| **Stroke** |  |  |  |  |  |
| Model 1 | Reference | 1.70（1.08-2.68） | 1.58（1.01-2.47） | 2.26（1.48-3.46） | 0.03 |
| Model 2 | Reference | 1.65（1.05-2.60） | 1.46（0.93-2.29） | 1.93（1.25-2.98） | 0.01 |
| Model 3 | Reference | 1.58（1.00-2.49） | 1.33（0.84-2.09） | 1.66（1.07-2.57） | 0.07 |
| **Ischemic stroke** |  |  |  |  |  |
| Model 1 | Reference | 1.96（1.15-3.35） | 1.63（0.95-2.80） | 2.61（1.57-4.32） | 0.01 |
| Model 2 | Reference | 1.89（1.11-3.24） | 1.49（0.87-2.56） | 2.14（1.28-3.57） | 0.01 |
| Model 3 | Reference | 1.79（1.04-3.06） | 1.33（0.77-2.30） | 1.80（1.07-3.03） | 0.10 |
| **Hemorrhage stroke** |  |  |  |  |  |
| Model 1 | Reference | 1.27（0.57-2.84） | 1.29（0.59-2.83） | 1.41（0.65-3.03） | 0.42 |
| Model 2 | Reference | 1.25（0.56-2.79） | 1.25（0.57-2.74） | 1.35（0.62-2.95） | 0.50 |
| Model 3 | Reference | 1.23（0.55-2.78） | 1.17（0.52-2.60） | 1.17（0.52-2.60） | 0.81 |

| **Continue Supplementary** **Table2** |  |  |  |  |  |
| --- | --- | --- | --- | --- | --- |
|  | **Quartile 1** | **Quartile 2** | **Quartile 3** | **Quartile 4** | ***P* for trend** |
| **Without Lipid-lowering treatment** |  |  |  |  |  |
| **Stroke** |  |  |  |  |  |
| Model 1 | Reference | 1.67（1.06-2.64） | 1.60（1.03-2.51） | 2.43（1.60-3.71） | <0.01 |
| Model 2 | Reference | 1.62（1.03-2.55） | 1.47（0.94-2.31） | 2.05（1.33-3.15） | 0.01 |
| Model 3 | Reference | 1.54（0.98-2.43） | 1.33（0.84-2.09） | 1.75（1.13-2.70） | 0.03 |
| **Ischemic stroke** |  |  |  |  |  |
| Model 1 | Reference | 1.92(1.12-3.29) | 1.68(0.98-2.87) | 2.89(1.75-4.77) | <0.01 |
| Model 2 | Reference | 1.85(1.08-3.16) | 1.51(0.88-2.59) | 2.32(1.40-3.87) | 0.01 |
| Model 3 | Reference | 1.74(1.01-2.98) | 1.34(0.78-2.31) | 1.96(1.17-3.28) | 0.03 |
| **Hemorrhage stroke** |  |  |  |  |  |
| Model 1 | Reference | 1.26(0.57-2.83) | 1.28(0.59-2.81) | 1.32(0.61-2.86) | 0.53 |
| Model 2 | Reference | 1.24(0.56-2.78) | 1.25(0.57-2.74) | 1.29(0.59-2.83) | 0.58 |
| Model 3 | Reference | 1.22(0.54-2.75) | 1.15(0.52-2.57) | 1.10(0.49-2.47) | 0.92 |

Model 1: adjusted for age and sex;

Model 2: adjusted for age, sex, smoking, drinking, education level, salt status, physical activity, and BMI;

Model 3: adjusted for all the variables in model 2 and LDL-C, HDL-C, hs-CRP, hypertension, antidiabetic drugs, antihypertensive drugs and lipid-lowering drugs.

TyG indicates triglyceride-glucose.

| **Supplementary Table 3** HRs for risk of outcomes after excluding the participants with subarachnoid hemorrhage stroke(n=11) | | | | | |
| --- | --- | --- | --- | --- | --- |
|  | **Quartile 1** | **Quartile 2** | **Quartile 3** | **Quartile 4** | ***P* for trend** |
| **Stroke, N（％）** | 23（0.26） | 55（0.61） | 64（0.71） | 128（1.42） |  |
| Incidence, per1000 person-y | 0.22 | 0.54 | 0.61 | 1.22 |  |
| Model 1 | Reference | 1.93（1.18-3.14） | 1.83（1.13-2.96） | 2.92（1.85-4.59） | <0.01 |
| Model 2 | Reference | 1.87（1.15-3.05） | 1.68（1.04-2.73） | 2.44（1.54-3.86） | 0.01 |
| Model 3 | Reference | 1.78（1.09-2.90） | 1.51（0.93-2.46） | 2.04（1.28-3.25） | 0.01 |
| **Ischemic stroke, N（％）** | 19（0.21） | 46（0.51） | 49（0.55） | 107（1.19） |  |
| Incidence, per1000 person-y | 0.19 | 0.45 | 0.47 | 1.02 |  |
| Model 1 | Reference | 1.96（1.14-3.37） | 1.70（0.98-2.93） | 2.96（1.76-4.96） | <0.01 |
| Model 2 | Reference | 1.89（1.10-3.25） | 1.54（0.89-2.64） | 2.40（1.43-4.03） | 0.01 |
| Model 3 | Reference | 1.78（1.03-3.07） | 1.36（0.79-2.36） | 1.98（1.17-3.35） | 0.02 |
| **Hemorrhage stroke, N（％）** | 5（0.06） | 11（0.12） | 15（0.17） | 23（0.26） |  |
| Incidence, per1000 person-y | 0.05 | 0.11 | 0.14 | 0.22 |  |
| Model 1 | Reference | 1.70（0.59-4.90） | 1.88（0.68-5.19） | 2.28（0.85-6.07） | 0.10 |
| Model 2 | Reference | 1.70（0.59-4.89） | 1.84（0.66-5.10） | 2.18（0.80-5.89） | 0.14 |
| Model 3 | Reference | 1.67（0.58-4.84） | 1.68（0.60-4.74） | 1.81（0.66-4.98） | 0.33 |

Model 1: adjusted for age and sex;

Model 2: adjusted for age, sex, smoking, drinking, education level, salt status, physical activity, and BMI;

Model 3: adjusted for all the variables in model 2 and LDL-C, HDL-C, hs-CRP, hypertension, antidiabetic drugs, antihypertensive drugs and lipid-lowering drugs.

TyG indicates triglyceride-glucose.

| **Supplementary Table 4** HRs from Competing Risk Models for outcomes after excluding the participants with subarachnoid hemorrhage stroke(n=11) | | | | | | |
| --- | --- | --- | --- | --- | --- | --- |
|  | **Total** | **Quartile 1** | **Quartile 2** | **Quartile 3** | **Quartile 4** | ***P* for trend** |
| **Stroke, N（％）** | 270 | 23（0.26） | 55（0.61） | 64（0.71） | 128（1.42） |  |
| Model 1 |  | Reference | 1.92（1.17-3.16） | 1.82（1.12-2.98） | 2.91（1.82-4.66） | <0.01 |
| Model 2 |  | Reference | 1.87（1.14-3.06） | 1.67（1.03-2.73） | 2.43（1.52-3.90） | <0.01 |
| Model 3 |  | Reference | 1.78（1.08-2.92） | 1.51（0.92-2.48） | 2.04（1.26-3.29） | <0.01 |
| **Ischemic stroke, N（％）** | 221 | 19（0.21） | 46（0.51） | 49（0.55） | 107（1.19） |  |
| Model 1 |  | Reference | 1.96（1.14-3.37） | 1.70（0.98-2.93） | 2.96（1.70-4.96） | <0.01 |
| Model 2 |  | Reference | 1.89（1.10-3.25） | 1.54（0.89-2.64） | 2.40（1.43-4.03） | 0..01 |
| Model 3 |  | Reference | 1.78（1.03-3.07） | 1.36（0.79-2.36) | 1.98（1.17-3.37） | 0.03 |
| **Hemorrhage stroke, N（％）** | 54 | 5（0.06） | 11（0.12） | 15（0.17） | 23（0.26） |  |
| Model 1 |  | Reference | 1.70（0.58-4.98） | 1.87（0.66-5.28） | 2.27（0.82-6.27） | 0.10 |
| Model 2 |  | Reference | 1.69（0.58-4.95） | 1.83（0.64-5.25） | 2.17（0.77-6.11） | 0.14 |
| Model 3 |  | Reference | 1.67（0.56-5.01） | 1.68（0.57-4.94） | 1.81（0.63-5.22） | 0.33 |

Model 1: adjusted for age and sex;

Model 2: adjusted for age, sex, smoking, drinking, education level, salt status, physical activity, and BMI;

Model 3: adjusted for all the variables in model 2 and LDL-C, HDL-C, hs-CRP, hypertension, antidiabetic drugs, antihypertensive drugs and lipid-lowering drugs.

TyG indicates triglyceride-glucose.
